# Structural and functional studies of a family of *Dictyostelium discoideum *developmentally regulated, prestalk genes coding for small proteins

**DOI:** 10.1186/1471-2180-8-1

**Published:** 2008-01-03

**Authors:** Juan J Vicente, María Galardi-Castilla, Ricardo Escalante, Leandro Sastre

**Affiliations:** 1 Instituto de Investigaciones Biomédicas, CSIC/UAM, C/Arturo Duperier, 4. 28029, Madrid. Spain

## Abstract

**Background:**

The social amoeba *Dictyostelium discoideum *executes a multicellular development program upon starvation. This morphogenetic process requires the differential regulation of a large number of genes and is coordinated by extracellular signals. The MADS-box transcription factor SrfA is required for several stages of development, including slug migration and spore terminal differentiation.

**Results:**

Subtractive hybridization allowed the isolation of a gene, *sigN *(SrfA-induced gene N), that was dependent on the transcription factor SrfA for expression at the slug stage of development. Homology searches detected the existence of a large family of *sigN*-related genes in the *Dictyostelium discoideum *genome. The 13 most similar genes are grouped in two regions of chromosome 2 and have been named Group1 and Group2 *sigN *genes. The putative encoded proteins are 87–89 amino acids long. All these genes have a similar structure, composed of a first exon containing a 13 nucleotides long open reading frame and a second exon comprising the remaining of the putative coding region. The expression of these genes is induced at10 hours of development. Analyses of their promoter regions indicate that these genes are expressed in the prestalk region of developing structures. The addition of antibodies raised against SigN Group 2 proteins induced disintegration of multi-cellular structures at the mound stage of development.

**Conclusion:**

A large family of genes coding for small proteins has been identified in *D. discoideum*. Two groups of very similar genes from this family have been shown to be specifically expressed in prestalk cells during development. Functional studies using antibodies raised against Group 2 SigN proteins indicate that these genes could play a role during multicellular development.

## Background

The social amoeba *Dictyostelium discoideum *is one of the simplest model systems utilized for the study of multi-cellular development. This organism lives as individual amoeba on forest soils, feeding on other microorganisms. However, when their food source is exhausted, they aggregate in groups of up to 100,000 cells and initiate a multi-cellular developmental program to form a fruiting body that stands on the substrate (reviewed in [1]). At the top of the fruiting body, inside the sorus, a large proportion of the original amoeba differentiate into resistant forms, called spores, that stay alive for long periods of time. Spores disseminate in the media and germinate to give rise to new amoeba when they reach favourable environmental conditions.

Aggregation of the amoebae is directed by chemotaxis to cAMP, secreted from discrete aggregation centres (reviewed in [2]). Cells that converge towards aggregation centres adhere among them forming small mounds covered by an extracellular matrix [3]. Cell-cell adhesion is mediated by several membrane proteins, whose expression is induced during development. The first known cell-cell adhesion system to be induced, soon after starvation, is Ca-dependent and is composed of the homophilic protein DdCAD-1 (gp24), encoded by the gene *cadA*[4]. A second homophilic, EDTA-resistant, adhesion system is induced at the onset of aggregation and is composed by the gp80 protein, encoded by the *csaA *gene [5,6]. A third adhesion system is induced later during aggregation being mediated by the gp150 proteins, encoded by the gene *lagC *[7]. Mutations in some of the genes coding for these adhesion systems or experimental conditions that interfere with their function, compromise the formation or stability of the multicellular structure [8-10].

Cells in the aggregates follow two alternative differentiation programs to become prestalk or prespore cells. At the same time, these cells continue to move towards cAMP secreted from the centre of the mound. Differences in chemotaxis to cAMP and in cell adhesion mediate the segregation of cell types so that prestalk cells migrate centrally and upwards to form a small protrusion, or tip, at the upper part of the structure [11]. This organization is maintained during most of development, including a migratory structure, the slug, that is formed under particular environmental conditions. Coordinated cell movement and differentiation continues during the rest of the morphogenetic process when prestalk cells move downwards to the substrate and differentiate to form the stalk. In the meanwhile, prespore cells migrate upwards to form the sorus where they differentiate to spores.

Morphogenesis is coordinated through the secretion of different factors that regulate cell adhesion, migration and differentiation, cAMP being the most important among them. The chlorinated alkyl phenone DIF-1 is also an important regulator of stalk cell differentiation [12]. Other cell-signalling factors are peptides or small proteins that are secreted by some cells to regulate the activity of neighbouring cells. For example, countin (258 amino acids long) and D11 (284 amino acids, encoded by the gene *ampA*) are secreted proteins that inhibit cell adhesion and contribute to regulate the size of the structure [13,14]. Also two secreted peptides, SDF-1 and SDF-2, originated by proteolysis of the AcbA protein, induce spore differentiation at culmination [15].

*Dictyostelium *multi-cellular development is also dependent on the co-ordinated regulation of gene expression. Multiple genes are either inhibited or induced at different stages of development [16]. This regulation is obtained by the activity of several transcription factors whose expression, or activity, is regulated during development [17]. One of the best-known transcription factors that regulates development is GBF (G-box binding factor), that is required for induction of many prestalk and prespore genes in the mound. For example, expression of the gene lagC, coding for the adhesion protein gp150, is dependent on GBF [18].

The MADS-box transcription factor SrfA is also involved in the regulation of multi-cellular development. Strains where the *srfA *gene has been knocked out showed defects in slug migration, morphogenesis and spore differentiation [19,20]. The identification of genes regulated by SrfA and involved in these processes can be an interesting approach to the study of development. Twenty four SrfA-dependent genes specifically expressed during spore differentiation have been described previously [21,22]. This article describes the isolation of SrfA-dependent genes expressed at the slug stage of development and focused on one of them, *sigN *(SrfA-induced gene N). This gene belongs to an extensive family of genes coding for small proteins, containing less than 100 amino acids, which are expressed in the prestalk region and seem to be involved in maintaining the integrity of the cell aggregates.

## Methods

### Cell culture, transformation and development

*Dictyostelium *cells were grown axenically in HL5 medium [23]. Transformations were carried out as described by Kuspa and Loomis [24]. Transformed cells were selected by treatment with blasticidine [25] or neomycin (GP418). Transformants were grown on SM plates in association with *Klebsiella aerogenes *for clonal isolation [23]. Development on nitrocellulose filters was performed as described by Shaulsky and Loomis [26].

### Subtractive library construction

RNA was isolated from slug structures of Wild Type (AX4) and *srfA*^- ^strains using Trizol reagent (Gibco-BRL). Four micrograms of poly(A)^+^RNA, purified using a mRNA purification kit (GEHealthcare), were used for synthesis of a cDNA subtraction library using a PCR-Select cDNA subtraction kit (Clontech), as previously described [21]. Wild-type cDNA was used as the tester cDNA, and *srfA*^- ^cDNA as the driver cDNA. Subtracted cDNAs were cloned in the pGEMT-Easy vector (Promega).

### Analyses of the promoter regions

Putative promoter regions of the *sigN2 *and *sigN9 *genes were amplified by PCR and cloned in the PsA-ialphagal vector [27], in substitution of the PsA promoter (excised as a XbaI-BglII fragment). Reporter vectors were transfected in AX4 cells by electroporation and the transformed colonies selected by geneticin (GP418) resistance. β-galactosidase activity was detected on developing structures as previously described [28].

### Generation of plasmid vectors

The plasmid vector used for RNA interference was based on exon 2 of *sigN3*. This exon was amplified by PCR and introduced in a pGEMT-Easy vector (Promega). This sequence was used to amplify the reverse complement. Forward and reverse fragments were cloned flanking a stuffer DNA [29] and the whole construct introduced in a *Dictyostelium *expression vector: pDXA-HC [30].

For generation of the *sigN *over-expression vector, a 300 bp fragment of the *sigN4 *gene was amplified by PCR and introduced in pGEMT-Easy vector. The fragment was taken out through a HindIII/XhoI digestion, and introduced in the expression vector pDXA-HC, under the control of the actin 15 promoter.

The *sigN *Group1 KO construct was generated from two regions of 460 and 1300 bp respectively, flanking the genomic region to be deleted. These fragments were amplified by PCR and introduced in the pBlueScript vector, flanking a blasticidin-resistance cassette. Oligonucleotides 5'-CGAATTCCCTTCTAATTGGTCTAATC-3' and 5'-CGCGGCCGCGGAGTTGGTTCCATTTTAACTGG-3' were used for amplification of the 460 bp fragment and oligonucleotides 5'-CGGATCCCGCCGTGGCGGCATTAGCATTAGC-3' and 5'-GAATTCGTGAGAACAGCACTGACTTACCTCC-3' for that of the 1300 bp fragment. The *sigN *Group 2 KO vector was constructed in a similar way from two fragments of 1000 and 730 bp, flanking the genomic region to be eliminated. These fragments were generated by PCR and cloned in pBlueScript at both sites of the Blasticidin-resistance cassette. Oligonucleotides 5'-CGAATTCCTACCGATGTTTCAGCAAGAGG-3' and 5'-CGGATCCCACAGTTGGTACCATTACAAATCC-3' were used for amplification of the 1000 bp fragment and 5'-CCGGTACCCTGTCAATGGAGTTGTTGGAGG-3' and 5'-CCCTGCAGGAATCAAGAGGATCTGGTCAATTGG-3' for that of the 730 bp fragment. The double-KO of Group 1 and Group 2 genes was made by co-transfection of the plasmid vector used for generation of the Group 1 deletion and the reporter vector ialphagal, carrying the neomycin resistance gene [27], in Group 2 mutant cells. Transformed cells were selected by treatment with GP418, grown on SM plates in association with *Klebsiella aerogenes *and individual colonies analysed for deletion of Group 1 genes by PCR. Colonies that presented deletion of Group 1 and Group 2 genes were isolated.

### Generation of antibodies against Group 1 and Group 2 proteins

The differences in amino acid sequence between group 1 and group 2 proteins were used to design group-specific peptides, CGSVLHGVGSILTGG (Group 1) and CGTVVGTVNGVVGGL (Group 2), used as antigens. Antibodies were generated by Genosphere Biotechnologies (Paris, France)

### Immunohistochemistry and Western blotting

Cells were washed free of liquid medium spread on cover slips and fixed with 4% PBS-paraformaldehyde during 30 minutes at room temperature. Mound structures were developed on Nitrocellulose Filters and transferred to cover slips before fixation. Cells and structures were washed twice with PBS, permeabilized with chilled methanol during 2 minutes, washed again with PBS and blocked with PBS+0,2% BSA for 20 minutes. Incubation with the first antibody was made in 150 μl of blocking buffer for 1 hour. Preparations were washed 6 times (5 minutes each) with PBS+BSA before incubation with150 μl of Alexa 568-coupled secondary antibody (dilution 1/1000) for 30 minutes. Preparations were washed twice with PBS+BSA and mounted with Prolong. Images were taken with a confocal microscopy (Olympus Fluoview 1000 confocal microscope) and processed with Adobe Photoshop software.

Western blot analyses of total protein extracts from vegetative cells were made as previously described [31].

### Sequence Alignments

Nucleotide and amino acid sequence alignments were performed with the ClustalW program at the San Diego Supercomputer Centre server. The alignments obtained were checked out in a local machine with the ClustalX program. Aligned sequences were used for the generation of phylogenetic trees using the CLC Free Workbench software.

## Results

### Identification of a large family of genes related to *sigN*, a *srfA*-dependent gene

Strains mutant for the *srfA *gene showed abnormal slug migration [20], suggesting that genes whose expression is regulated by the transcription factor SrfA could be directly involved in proper function of the slugs. The isolation of SrfA-dependent genes was approached by the synthesis of a differential cDNA library using RNA isolated from WT slugs subtracted with RNA from *srfA*^- ^slugs. Several cDNA clones were obtained whose expression was higher in WT than in *srfA*^- ^slugs. One of them, named *sigN *(SRF-induced gene N), is described in this article.

**Figure 1:**
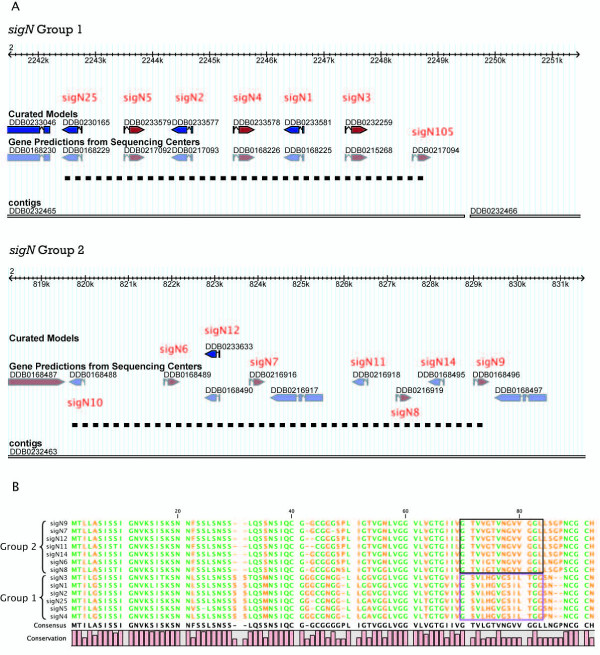
**Chromosomal organization and similarity of Groups 1 and 2 *sigN *genes**. Panel A schematically shows the location of Group 1 (upper part) and Group 2 (lower part) genes in chromosome 2. Exon coding regions are indicated as boxes and intron sequences as lines. Direction of transcription is indicated by arrowheads. Genomic fragments (contigs) where the genes are located, and the database number (curated and gene prediction) for each gene are shown in the figure. The position of exon 1 of the *sigN2 *gene was modified with respect to the curated model after re-sequencing of this genomic region. Dashed lines represent the genomic regions eliminated in KO strains; from bp 819699 to 829288 for Group 2, and from bp 2242478 to 2248753 for Group 1. B. Protein sequences deduced for *sigN *genes were compared trough the use of clustal alignments. Amino acid identity is represented as bars (conservation). Amino acids conserved in all proteins are represented in green. The more divergent regions, used to generate antibodies specific for the proteins of each group are boxed.

The nucleotide sequence of the *sigN *cDNA was compared with the *D. discoideum *genome sequence. The corresponding gene is located in chromosome 2 and contains two exons separated by one small intron. The open reading frame region contained in exon 1 is 13 nucleotides long, and that of exon 2 is 254 nucleotides long. The encoded protein is, therefore, 89 amino acids long and contains a large proportion of serine cystein and glycine residues (Fig 1B). Blast searches of *Dictyostelium *SigN protein did not detect any homology with proteins in other organisms. The product of the gene *sigN*, appears in the *Dictyostelium *database as a "cysteine knot domain-containing protein". Cysteine-knot is a special folding domain found in many extracellular proteins like TGF-β2, PDGF, NGF and some neurotoxins. This kind of structure involves six conserved cysteines, generating three intramolecular disulphide bridges arranged in a knot-like topology, which gives a highly efficient motif for structure stabilization. The difference between this classic cysteine knot and the knot found in SigN1 is the number of cysteines, four instead of six, in this hypothetical protein. Several programs also predicted the existence of a putative transmembrane domain between amino acids 54 and 76 of the protein.

**Figure 2:**
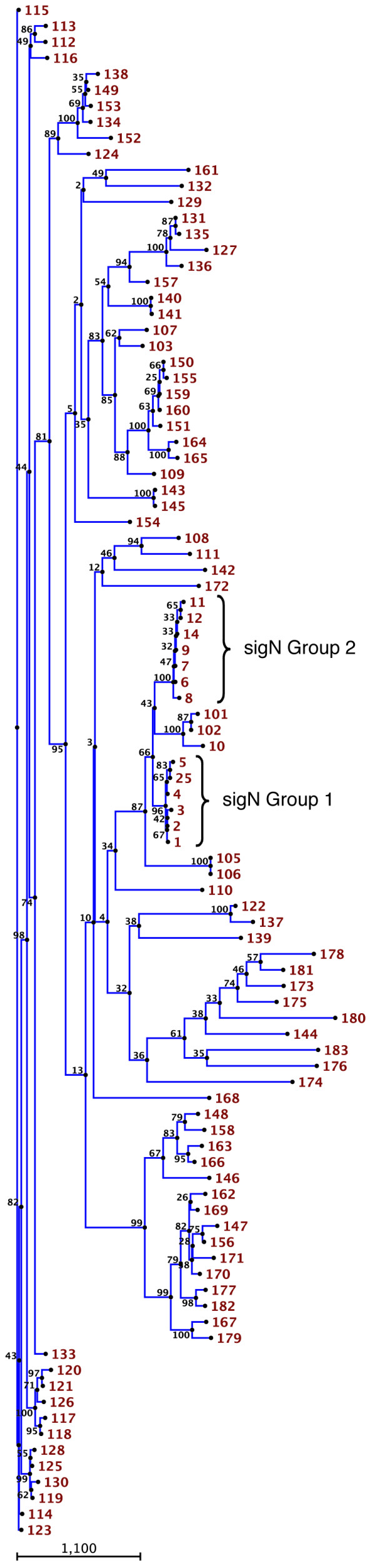
**Phylogenetic tree of the proteins encoded by the family of *sigN *genes**. The predicted amino acid sequences encoded by 96 genes that showed significant similarity to *sigN1 *were compared using the ClutalW program at the online Biology WorkBench facilities from San Diego Supercomputer Center. The alignment was used to construct a phylogenetic tree using the neighbor-joining method and 100 bootstrap trials. The number of times each branch was obtained is indicated. The number assigned to each *sigN *gene is shown to the right. Group 1 and Group 2 *sigN *genes are indicated by brackets.

Homology searches in the Dictyostelium genome detected the existence of 96 genes coding for small proteins (less than 120 amino acids) with high similarity to SigN. Most of the genes (89/96) presented the same gene structure: one exon with a 13 nt long open reading frame, one intron and a second exon. The closest homologous genes are shown in Table 1 and a complete relation is shown in Additional file 1. The phylogenetic relation among these proteins is shown in Figure 2. The analyses of functional domain databases showed a hypothetical "coiled-coil" secondary structure in some of the proteins. This is a structural domain usually involved in oligomerization of proteins and is present in proteins with very different functions, which made it impossible to predict *a priori *a possible function for these proteins.

**Table 1:** *Dictyostelium discoideum *genes highly similar to *sigN1*.

**Gene Name**	**DDB (prediction)**	**DDB (curated)**	**Chromosome**	**Exons number – size (bp)**	**Intron size (bp)**	**Aminoacids**	**Identity with sigN1 protein (%)**
*sigN1*	DDB0168225	DDB0233581	2	1 – 13//2 – 256	66	89	100
*sigN2*	DDB0217093	DDB0233577	2	1 – 13//2 – 256	70	89	99
*sigN3*	DDB0215268	DDB0232259	2	1 – 13//2 – 256	107	89	95
*sigN4*	DDB0168226	DDB0233578	2	1 – 13//2 – 256	82	89	95
*sigN25*	DDB0168229	DDB0230165	2	1 – 13//2 – 253	71	88	95
*sigN5*	DDB0217092	DDB0233579	2	1 – 13//2 – 250	89	87	94
*sigN7*	DDB0216916		2	1 – 13//2 – 253	79	88	69
*sigN12*	DDB0168490	DDB0233633	2	1 – 13//2 – 253	72	88	68
*sigN14*	DDB0168495		2	1 – 13//2 – 256	71	89	68
*sigN6*	DDB0168489		2	1 – 13//2 – 256	79	89	67
*sigN9*	DDB0168496		2	1 – 13//2 – 256	79	89	67
*sigN8*	DDB0216919		2	1 – 13//2 – 256	80	89	66
*sigN11*	DDB0216918		2	1 – 13//2 – 253	79	88	65
*sigN10*	DDB0168488		2	1 – 13//2 – 256	84	89	62
*sigN105*	DDB0217094	DDB0238206	2	1 – 13//2 – 214	87	75	62
*sigN103*	DDB0191897	DDB0230164	6	1 – 13//2 – 268	89	93	41
*sigN107*	DDB0191896	DDB0231563	6	1 – 13//2 – 268	111	93	35
*sigN110*	DDB0168566		2	1 – 13//2 – 250	110	87	33

Analysis of the complete sequence of *Dictyostelium *genome at the DictyBase database allowed determination of the location of all these genes. The gene coding for the cDNA initially isolated and the closest homologous genes were grouped in a 7 kb long region of chromosome 2 (Fig 1A and Table 1). All these genes code for highly similar proteins (Fig 1B, Fig 2) and have been named *sigN1 *(the gene isolated from the subtractive library), *sigN2*, *3*, *4*, *5 *and *25 *(Group 1). Another group of genes with high identity to *sigN1 *was found in a different 10 kb long region of chromosome 2 (Fig 1A). The members of this second group showed lower identity with *sigN1*, between 60–70% of the amino acids of the encoded proteins are identical to SigN1 (Table 1). However, the identity among the proteins encoded by this second group of genes was about 95% (Fig 1B), which made them group together in the phylogenetic tree (Fig 2). These genes have been named *sigN6*, *7*, *8*, *9*, *11*, *12 *and *14 *(Group 2). The identity of the proteins encoded by the two groups of genes was very high in their N-terminal regions. However, there were some group-specific differences at the C-terminal region (Fig 1B). Genes DDB0217094 (*sigN105*) and DDB0168488 (*sigN10*) are located in the chromosomal region of group 1 and 2 genes, respectively. They have not been included in these groups because of their lower identity with the other genes, as shown in the phylogenetic tree (Fig 2). The identity of the hypothetical protein encoded by *sigN105 *with group 1 proteins was about 62% and that encoded by *sigN10 *showed an identity with the members of group 2 of about 64%.

### Temporal expression of *sigN *genes

**Figure 3:**
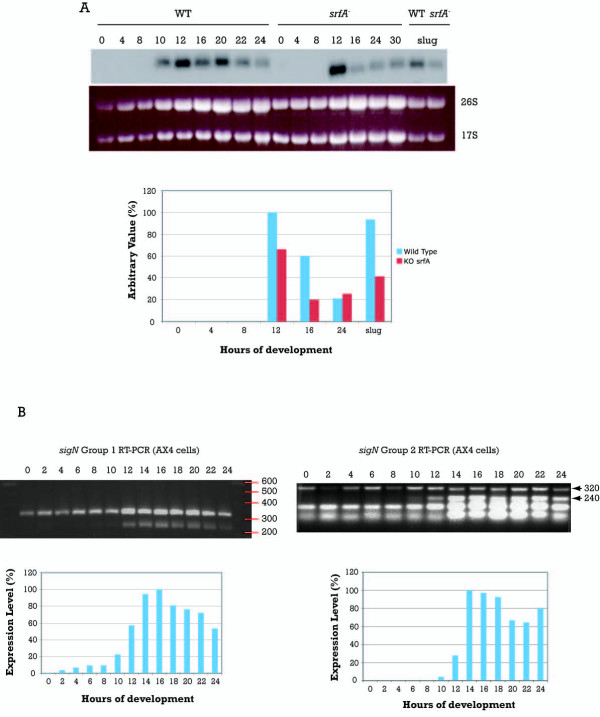
**Expression pattern of *sigN *genes during multicellular development**. A. RNAs from WT and *srfA*^- ^strains were collected at the indicated hours (0–30) or at the slug stage of development (slug). The coding region of the *sigN1 *gene was used as probe for hybridization. The mRNA detected migrated faster than the 18S rRNA. Ethidium bromide staining of the gel is shown in the lower panel. Hybridization signals were quantified by densitometry and normalized for the rRNA present in each lane (lower graphic). The value obtained for the 12 hours sample of the WT strain was considered 100% of expression. B. RNA was obtained from WT (AX4) strains, collected at the indicated hours of development. *SigN *genes expression was determined by RT-PCR using oligonucleotides specific for each group of genes. The sequence amplified for both groups was about 240 bp, and the control sequence, obtained by amplification of a region of the large mitochondrial ribosomal RNA, about 320 bp. Bands were quantified by densitometry, normalized in relation to the control and represented in a bar graphic, assigning 100% expression to the16 hours sample for Group 1 and 14 hours sample for Group 2.

The cDNA isolated in the differential screening was used as a probe to analyze the developmental expression of these genes in WT and *srfA*^- ^strains by Northern blot (Fig 3A). No expression was detected in proliferating cells (time 0 of development) or during the first hours of development. *sigN *expression was induced at 10 hours in WT cells and is maintained during the rest of development. SrfA mutant strains also showed induction of *sigN *expression at 12 hours of development but gene expression was lower than in WT cells, especially at later stages of development. The expression of *sigN *at the slug stage of development was also lower in the *srfA*^- ^strains than in WT, in agreement with the results obtained in the subtractive method used for isolation of the gene.

The probe used to determine the differential expression of *sigN *covered 190 nt in the coding sequence of this gene. It is very likely that the probe can detect Group 1 and Group 2 mRNAs, due to the high identity that exists among the genes. Specific oligonucleotides were designed to study the expression of the two groups of genes independently, based on the differences in their C-terminal-coding regions. RT-PCR studies showed that expression of Group 1 genes was first detected at 2 hours of development with a rise at 12 hours, when the mound is completely formed (Fig 3B). Group 2 genes showed no expression until 10 hours of development with maximal induction obtained at 14 hours. In this case, the lower bands obtained in the RT-PCR reactions were sequenced and did not correspond to any specific amplification product.

### Analyses of *sigN *promoters

**Figure 4:**
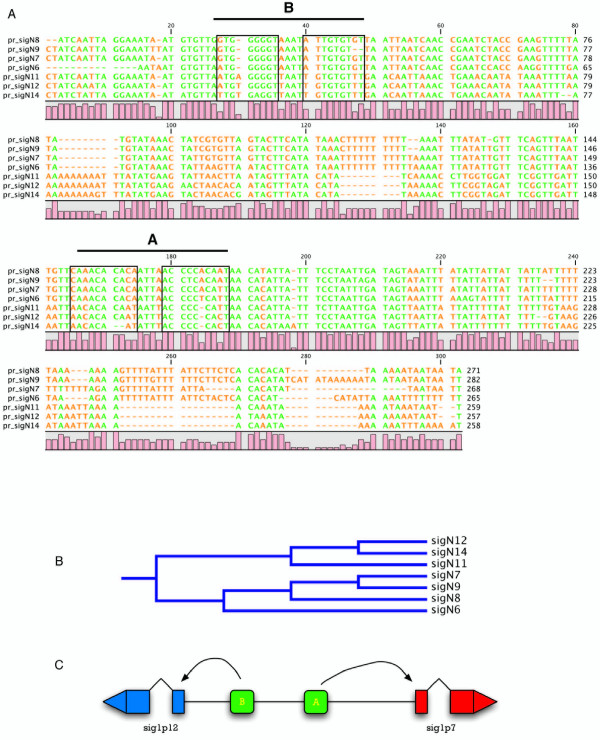
**Similarity of the putative promoters regions of *sigN *Group 2 genes**. A. The sequences of the putative promoters of *sigN *Group 2 genes, considered as the upstream regions from the initiation codon to the closest gene-coding region, were compared and the alignment of the regions containing putative regulatory sites is shown. The boxes in the figure indicate the possible binding sites for the transcription factor GBF (G-box Binding Factor). Pairs of contiguous binding sites have been grouped and labelled as regulatory regions A and B. Identical nucleotides are indicated in green. The level of identity between nucleotides of the different genes is represented as bars (conservation). B. Phylogenetic tree calculated from the nucleotides alignments shown in panel A using the Clustal W program. C. Schematic representation of the location of conserved, potential regulatory regions (A and B) present in the intergenic region between each pair of inverted genes, represented as rounded boxes. Coding regions are represented as boxes and intron and untranslated regions as lines. The direction of transcription of each gene is represented by arrow heads.

The temporal pattern of expression of the *sigN *genes and their partial dependence on the SrfA transcription factor prompted the interest in the study of their promoter regions. The complete intergenic region upstream of each gene, up to the closest coding region, was considered putative promoter region in these studies. In some cases, genes are adjacent and in opposite orientation (For example, genes *sigN1 *and *sigN3 *in Fig 2A) and their putative promoter regions are complementary to each other. Clustal alignment studies were performed to find possible identities among putative promoter regions. The intergenic regions of Group 2 genes showed a high sequence identity (Fig 4A). The similarities of the putative promoter regions are indicated in the phylogenectic tree shown in Figure 4B. Promoter regions of the genes *sigN7 *and *9 *(and their complementary sequences *sigN12 *and *14*) are more similar between them and slightly more distant from *sigN8 *(complementary to *sigN11*) and *sigN6*. Putative promoter regions of adjacent genes, that are inverted in relation to each other, also shown significant identity among them indicating their palindromic structure, as schematically shown in Figure 4C. These data indicate that the genes in this group could have derived by successive duplication of a tandem of two inverted *sigN *genes.

Intergenic regions of the Group 1 genes *sigN1*-*sigN3 *and *sigN2*-*sigN4 *are very similar between them but do not have the palindromic structure described for Group 2 genes. Therefore, the putative promoter region of the *sigN1 *gene is very similar to that of *sigN2 *and the same hold true for *sigN3 *and *sigN4 *regions. However, the *sigN1 *promoter region does not show similarity to that of the contiguous *sigN3 *gene. Similarly, the *sigN2 *putative promoter region does not show similarity to that of *sigN4*. These regions do not show similarity with the sigN5 and sigN25 putative promoter regions, either.

The comparison of the nucleotide sequence of the putative promoter regions of Group2 genes detected the existence of four repetition of a conserved G-rich sequence (boxed in Fig 4A), also present in Group 1 promoters. These sequences are very similar to the GBF response element: (T/G)G (T/G)G (T/G)G (T/G) [32]. Group 2 promoter regions contain two pairs of closely locate GBF-binding sites, labelled as regions A and B in Figures 4A and 4C, that could regulate the expression of the contiguous opposite genes.

**Figure 5:**
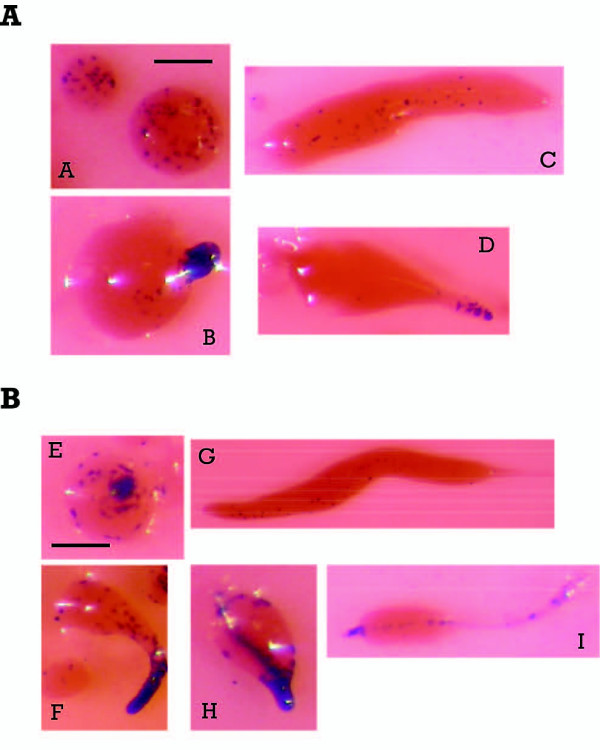
**Cell-type specific activity of *sigN *gene promoters**. A. AX-4 cells were transfected with a reporter vector driving *lacZ *expression under the control of the Group 1 *sigN2 *promoter. β-galactosidase activity, detected by X-gal staining, is shown in structures at mound (A), finger (B), slug (C) and early culminant (D) structures (Bar, 0,1 mm). B. A reporter vector where lacZ was placed under the transcriptional control of the Group 2 *sigN9 *promoter was transfected in AX-4 cells. X-gal staining is shown for mound (E), finger (F), slug (G), early-culminant (H) and culminant (I) structures (Bar, 0,1 mm).

The functionality of the putative promoter regions was tested using reporter vectors where a *lacZ *gene coding for unstable β-galactosidase was placed under the transcriptional control of *sigN *promoters. These assays also allowed determining the spatial pattern of expression of these genes during development. The high similarity of these genes made very difficult the use of *in situ *hybridization to study the expression of specific genes of this family during development. These studies were focused on putative promoter regions of one Group 1 gene (*sigN2*) and one of the Group 2 genes (*sigN9*) given the similarity observed between the promoters of each group. Staining of the structures transformed with the reporter vectors indicate that both putative promoter regions direct gene expression in the prestalk region of developing structures (Fig 5). Promoter of the *sigN2 *gene showed weaker activity in these assays but *lacZ *expression was first observed in disperse cells in the mound stage of development. Later on, prestalk cells placed at the tip of the structure showed *lacZ *expression in first finger and early culminant structures (Fig 5A). At the slug stage promoter activity was detected in cells dispersed through the structure, with a pattern similar to that of Anterior Like Cells (Fig 5A). The promoter of the *sigN9 *gene also directed expression in dispersed cells in mounds and in prestalk cells at the tip of first finger and early culminant structures and in dispersed cells in the slugs (Fig 5B). In addition, *sigN9 *promoter also directed lacZ expression in the cells that migrate from the tip to form the stalk during culmination and in stalk cells of the fruiting body.

### Function of *sigN *genes

**Figure 6:**
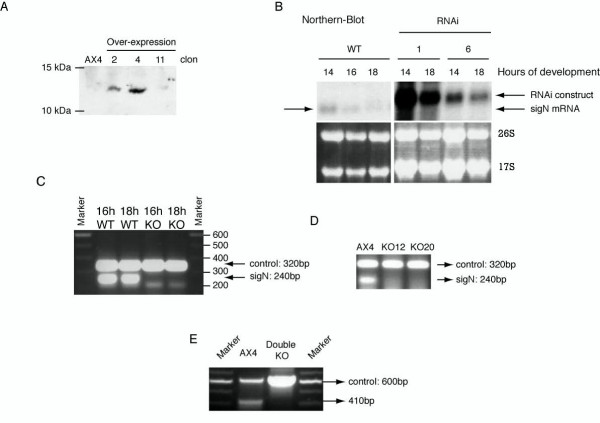
**Characterization of strains where *sigN *genes have been over-expressed, repressed by the use of interfering RNA or deleted**. A. Total protein extracts were obtained from WT (AX4) and three strains (2, 4, 11) transfected with a plasmid vector for *sigN4 *over-expression. Group 1 antibodies were used for Western-Blot. The migration of the 10 and 15 kDa protein markers is shown on the left. B. RNA was obtained from WT and two different RNAi clones that expressed an interfering RNA based on exon 2 of *sigN3 *(1 and 6 clones) after 14–18 hours of development on Nitrocellulose filters. Arrows indicate the position of the endogenous mRNA (*sigN *mRNA) and of the RNAi transcribed from the vector (RNAi construct). Ethidium bromide staining of the gel is shown in the lower panel. C. RNA from WT (AX4) and Group 1 KO strains, harvested at the indicated hours of development (16–18 h) was analyzed by RT-PCR for expression of *sigN *Group 1 genes. The control band obtained by amplification of a region of the large mitochondrial ribosomal RNA (control: 320 bp long) and that corresponding to *sigN *Group 1 mRNA (*sigN*:240 bp) are indicated by arrows at the right. D. RNA was obtained from WT (AX4) and two different Group 2 KO strains (KO12, KO20) after 16 hours of development. Expression of *sigN *Group 2 genes was analyzed by RT-PCR. The migration of the control band (control: 320 bp) and that corresponding to *sigN *Group 2 mRNA (*sigN*: 240 bp) is indicated at the right. E. DNA from WT (AX4) and a Group 1 and Group 2 Double KO strain (Double KO) was analyzed by PCR for the presence of for *sigN *Group 1 genes. Migration of the amplified fragments for a control gene (control: 600 bp) and *sigN *Group 1 genes (410 bp) is indicated at the right.

Over-expression and under-expression of *sigN *genes was intended to study the function of these genes. Over-expression was performed by cloning the gene *sigN4 *under the control of the actin 15 promoter. Western blot analyses of vegetative cells using antibodies specific for Group 1 proteins indicated the expression of a 12 kDa protein in the over-expressing strains that was not detected in the WT strain (Fig 6A). No difference in phenotype was found between the WT and the over-expression strains. Downregulation was initially approached by the expression of interfering RNA. Two copies of *sigN3 *exon 2 were placed in an inverted orientation, separated by a stuffer sequence, under the control of an actin 15 promoter. The great identity in the N-terminal-coding region of these genes, could make possible that the iRNA fragments generated from this construct would interfere with the expression of all *sigN *genes. Strains expressing this iRNA construct showed a strong reduction in the expression of sigN RNAs (Fig 6B) but did not show any difference in their phenotype in relation to WT strains. Mutant strains were also obtained where all *sigN *genes of Group1, Group2 or both groups were deleted by homologous recombination. Deleted regions are indicated in Figure 1A. RT-PCR analysis was used to corroborate the absence of *sigN *expression in these strains (Fig 6C–E). However, neither of the mutant strains did show any difference in growth or development in comparison to the WT strain.

**Figure 7:**
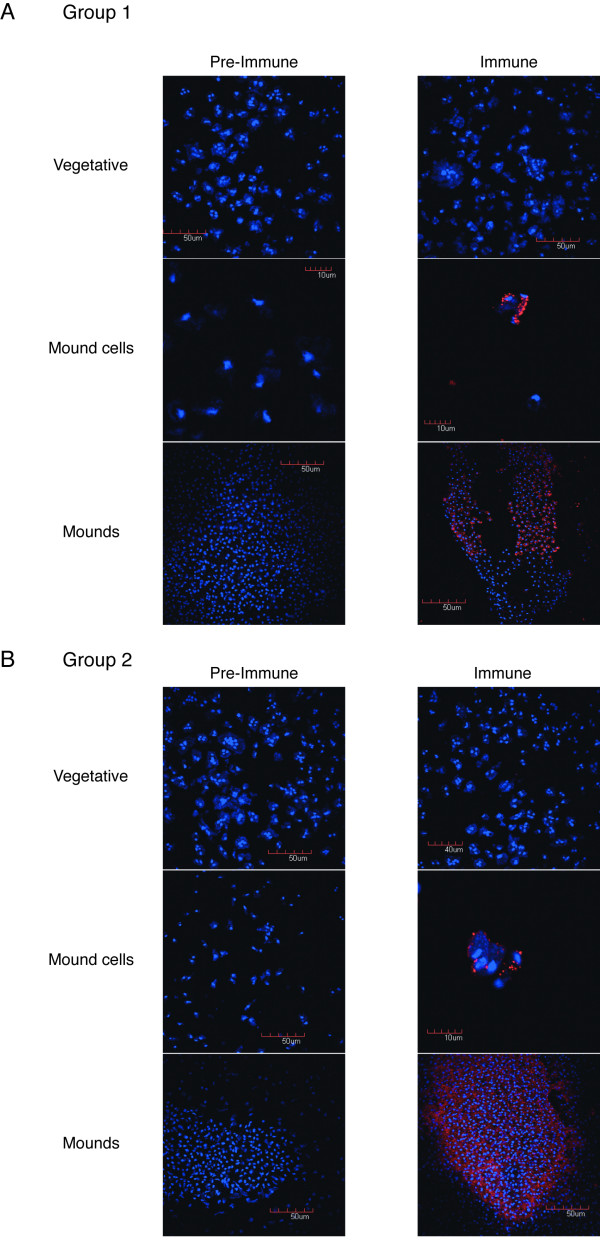
**Immunocyotchemical analyses of SigN expression**. Vegetative cells (vegetative), cells obtained by dispersion of mound structures developed for 14 hours (Mound cells) or mound structures (Mounds) were fixed and stained with preimmune serum (Pre-immune) or serum raised against specific peptides (Immune) corresponding to Group 1 (upper panels) or Group 2 (lower panels) genes Alexa 568-conjugated anti-rabbit serum was used as secondary antibody. DAPI was used for nuclear staining.

Many proteins are secreted during *Dictyostelium *development to coordinate morphogenesis and differentiation. Some of these proteins have low molecular weight and present post-translational modifications, like glycosylations. Examples of these molecules are PSF, CMF, countin factor and SDF, as mentioned in the Introduction section. The small size of SigN proteins, the existence of hypothetical glycosylation sites and the predicted presence of a cysteine-knot structure and a putative transmembrane region would be in agreement with an extra-cellular function for these proteins. To test this hypothesis, specific antibodies were raised against the proteins of each group. Immunocytochemical analysis using these antibodies detected staining in small vesicles present in a fraction of the cells obtained after dispersion of the cells from mound structures obtained after 14 hours of development (Fig 7). Staining was not observed in vegetative cells, using immune or preimmune serum, or in mound cells using pre-immune serum (Fig 7). Staining in vesicles would be in agreement with the hypothesis that Group 1 and Group 2 proteins are secreted to the extra-cellular medium.

To further study the possible function of these proteins, antibodies were added in vivo to developing cells to try interfering some of the developmental processes. Pre-immune antiserum was used as control. Specific antibodies against Group 1 proteins did not show any obvious interference with the developmental process in comparison with pre-immune and phosphate-based buffer (PDF) controls.

**Figure 8:**
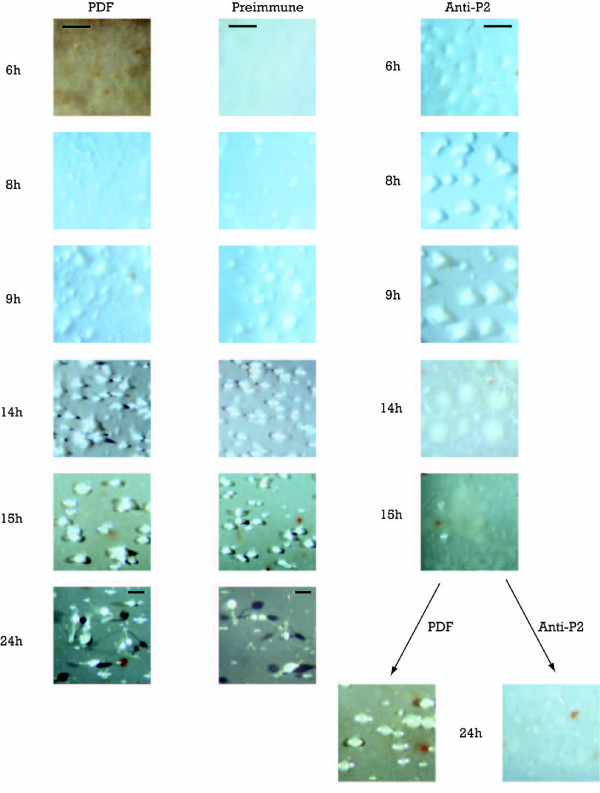
**Development in the presence of antibodies specific for Group 2 *sigN *proteins**. AX4 cells were placed on nitrocellulose filters to induce multicellular development. Filters were placed over pads soaked on phosphate buffer alone (PDF) or containing preimmune serum (preimmune) or a serum raised against a Group 2-specific peptide (Anti-P2). At 15 hours of development, the antiserum filter was divided in two parts. One half was placed on a new pad soaked in PDF alone (PDF) and the other on a pad soaked in PDF containing Group 2 antiserum (Anti-P2), as shown in the lower part of the right-hand panels. Pictures were taken at the times of development indicated at the left of the panels with a stereomicroscope (Bar, 0,15 mm).

The addition of antibodies specific for the Group 2 proteins produced some differences, in comparison to pre-immune or PDF controls. Mounds were formed two hours earlier in the presence of Group2-specific antibodies. From there on, mounds began to disaggregate and, at 15 hours of development, when a finger is normally formed, no visible structure remained in the filters (Fig 8). At 15 hours of development the filter treated with the antibody was divided in two parts. One of them was incubated for 10 more hours with a PDF phosphate-based buffer, and the other with antibody added to fresh medium. The part incubated in PDF developed in a usual way, forming new mounds. The part of the filter that continued in the presence of the antibody was unable to form any structure (Fig 8). These results make us suggest that *sigN *Group 2 genes could be involved in the aggregation process, contributing to maintain the coherence of the multi-cellular structure.

## Discussion

The study of the gene *sigN1*, partially dependent on the transcription factor SrfA for expression, has uncovered the existence of a family of genes coding for very similar small proteins in *D. discoideum*. The family is large with, at least, 96 genes, although the present study has been centred on 13 closely related genes located in two regions of chromosome 2. These genes have in common their structure and developmental pattern of expression, in addition to the high similarity of their nucleotide sequences. These genes have been divided in two groups based on the sequence of a small divergent region that code for the C-terminal region of the protein, and their chromosomal location. Phylogenetic analyses of the coding and intergenic regions suggest that the genes of each group might have originated by duplication of an original tandem of two genes, oriented in opposite directions.

All the genes are constituted by an exon, containing a 13 nt long open reading frame, an intron and a second exon, coding for the rest or the protein. The same structure is share by most of the 96 similar genes mentioned above. The conserved structure of these genes might be indicative of their common evolutionary origin.

Northern and RT-PCR studies have shown that Group1 and Group2 *sigN *genes expression is induced at 10–12 hours of multi-cellular development. Analyses of the promoter regions of one gene of each group indicate that *sigN *genes are expressed in the prestalk region. The sequence of the putative promoter regions, very similar among Group 2 genes, contain several copies of a conserved G/C rich motif similar to the GBF transcription factor binding site [32]. GBF expression is developmentally regulated and necessary for the expression of numerous prestalk and prespore genes [10]. Microarray analysis of GBF-dependent genes identified *sigN12 *as one of them [18], in agreement with the presence of putative GBF-binding sites in the promoter region. Two other *sigN *related genes, DDB0230164 (*sigN103*) and DDB0231563 (*sigN107*), coding for proteins that are 41% and 35% identical to SigN1, respectively, are also dependent on GBF for their expression The developmental pattern of expression of these genes was also very similar to that of *sigN *genes. Besides, Microarray and in situ hybridization analyses of the expression of these genes have shown that these three GBF-dependent genes are expressed in the prestalk region, including *sigN12 *[18,33]. These data are in agreement with the results presented in this article and suggest that, since all these genes are very similar in sequence and present similar temporal and spatial patterns of expression, might accomplish similar, and perhaps redundant, biological functions.

The study of the function of *sigN *genes was approached by their over-expression, under-expression and by deletion. However, even the deletion of the 13 Group 1 and Group 2 genes seemed to have no effect on development. These data could be explained by the redundant functions of this family of genes, as previously suggested on the bases of their structure and expression.

Only the addition of specific antibodies raised against Group 2 *sigN *genes had some effect on development, inducing disaggregration of mound structures. This effect was specific since antibodies raised against Group 1 peptides or pre-immune serum had no effect on this process. With the exception of the *sigN4 *over-expressing strain, the antibodies raised against SigN peptides did not recognize these proteins in Western blot, which precluded further studies on their specificity. With this caveat in mind, the simplest explanation for these results obtained would be that Group 2 SigN proteins could participate in cell-cell adhesion and that the presence of the antibody could block interactions of SigN with other proteins, required for cell adhesion. As mentioned in the Results section, many proteins containing cysteine knot domains are extracellular, which would be in agreement with the function proposed for SigN Group 2 proteins. Antibodies raised against Group 1 and Group 2 proteins stained small vesicles in cells at the mound stage or development, which would be also in agreement with their proposed secretion. The expression pattern was similar for both antibodies, as expected from the similar structural domains predicted for the proteins of both groups.

According to the hypothesis suggested above, *sigN *Group 2 mutants would be expected to show defective aggregation, which was not the case. As mentioned above, functional redundancy between *sigN *genes could explain these results since the sig *N *Group 2 mutants could still maintain cell adhesion through the interaction of other related proteins. A search for proteins containing sequences similar to the one of the peptide used to raise Group 2 specific antibodies showed that at least two other proteins of the *sigN *family, encoded by genes DDB0230164 (*sigN103*) and DDB0168566 (*sigN110*), presented sequences very similar to the peptide. One of these genes, DDB0230164 (*sigN103*), was also dependent on GBF for expression. It is, therefore, possible that these proteins could mediate cell adhesion in *sigN *Group 2 mutants. Antibodies would bind to all the proteins that contain this sequence, including Group 2 and other proteins, blocking all protein interactions. In agreement with this hypothesis, the antibody is able to disintegrate mounds formed by the Group 2 mutant strain (data not shown). These results are similar to those previously described for other cell-cell adhesion proteins. For example, a peptide from the N-terminal region of the gp80 protein prevented cell adhesion [9] while a mutant lacking the encoding gene (csA) had no obvious phenotype in experimental conditions similar to the ones used in the present study [34]. Antibodies specific for the cell adhesion protein DdCAD-1 also arrested development at aggregation [35].

SigN Group 2 proteins could be also involved in the formation of the extracellular matrix that surrounds multi-cellular structures, isolating them from the environment and providing a substrate for cell adhesion and migration. The extracellular matrix is composed of cellulose and proteins, synthesized by prestalk cells [3]. Early studies on the composition of the extra-cellular matrix identified a population of urea-insoluble proteins smaller than 15 kDa in size. These proteins had an higher content in serine, cysteine, glycine, valine and aspartic acid/asparagine and a lower content in lysine and arginine than whole-cell proteins [36]. SigN proteins had a similarly high proportion of cysteine, glycine and serine and a low proportion of lysine and arginine. Besides, they are expressed in prestalk cells, which would be compatible with their presence in the extra-cellular matrix.

Immunocytochemical studies detected the presence of these proteins in small vesicles of mound cells. However, no staining was found either at the plasma membrane, as expected if they were involved in cell-cell interactions, or at the extracellular matrix, making impossible to discern between the alternative possibilities discussed above. The lack of reactivity could be due to conformational changes in the extra-cellular medium or to their incorporation into structures where they would not be accessible to the antibodies used in immunocytochemistry. Therefore, while a function in cell adhesion or extracellular matrix formation is suggested, the lack of information on the specificity of the antibodies and a inability to detect membrane or external antibody binding necessitates further experiments to support this conclusion.

## Conclusion

A large family of genes coding for small proteins has been identified. Most of the genes have a similar structure containing a first exon, coding for a 13 nucleotide long open reading frame region, an intron, and a second exon coding for the rest of the protein. Two groups of these genes, coding for very similar proteins and closely assembled in two different regions of chromosome 2, have been studied in more detail. These genes are specifically expressed at the prestalk region during development of multicellular structures. Immunochemical studies indicated that these proteins could be secreted. The addition of antibodies raised against group 2 proteins avoided formation of cellular aggregates, which suggest a possible role for these proteins in development.

## Authors' contributions

JJV carried out most of the experimental work, the analyses of the data and helped in the design of the experiments and to draft the manuscript. MG-C carried out the analyses of the gene promoter regions. RE participated in the design of the study and the analyses of the results. LS participated in the design and coordination of the study and draft the manuscript. All authors read and approved the final manuscript.

## Supplementary Material

Additional file 1 Structure and chromosomal location of *Dictyostelium *genes similar to *sigN1*. In this table the DictyBase database accession number, chromosomal location and exon/intron structure of the genes similar to sigN1 is indicated together with the size of the encoded proteins and their percentage of similarity to SigN1. Click here for file
