# Supplementary material for: Structural and functional studies of a family of Dictyostelium discoideum developmentally regulated, prestalk genes coding for small proteins
Source: BMC Microbiol. 2008 Jan 3;8:1. doi: 10.1186/1471-2180-8-1 (PMC2257962; doi:10.1186/1471-2180-8-1)
Supplement: Additional file 1 — Structure and chromosomal location of Dictyostelium genes similar to sigN1. In this table the DictyBase database accession number, chromosomal location and exon/intron structure of the genes similar to sigN1 is indicated together with the size of the encoded proteins and their percentage of similarity to SigN1. [file 1471-2180-8-1-S1.pdf]

Gene Name	DDB (prediction)	DDB (curated)	Chromosome	Exons (exon number - bp)	Introns (intron number - bp)	Aminoacids	Identity with sigN1 protein (%)
sigN1	DDB0168225	DDB0233581	2	1 - 13 // 2 - 256	1 - 66	89	100
sigN2	DDB0217093	DDB0233577	2	1 - 13 // 2 - 256	1 - 70	89	99
sigN3	DDB0215268	DDB0232259	2	1 - 13 // 2 - 256	1 - 107	89	95
sigN4	DDB0168226	DDB0233578	2	1 - 13 // 2 - 256	1 - 82	89	95
sigN25	DDB0168229	DDB0230165	2	1 - 13 // 2 - 253	1 - 71	88	95
sigN5	DDB0217092	DDB0233579	2	1 - 13 // 2 - 250	1 - 89	87	94
sigN7	DDB0216916		2	1 - 13 // 2 - 253	1 - 79	88	69
sigN12	DDB0168490	DDB0233633	2	1 - 13 // 2 - 253	1 - 72	88	68
sigN14	DDB0168495		2	1 - 13 // 2 - 256	1 - 71	89	68
sigN6	DDB0168489		2	1 - 13 // 2 - 256	1 - 79	89	67
sigN9	DDB0168496		2	1 - 13 // 2 - 256	1 - 79	89	67
sigN8	DDB0216919		2	1 - 13 // 2 - 256	1 - 80	89	66
sigN11	DDB0216918		2	1 - 13 // 2 - 253	1 - 79	88	65
sigN10	DDB0168488		2	1 - 13 // 2 - 256	1 - 84	89	62
sigN102	DDB0185643		4	1 - 13 // 2 - 247	1 - 66	86	62
sigN105	DDB0217094	DDB0238206	2	1 - 13 // 2 - 214	1 - 87	75	62
sigN106	DDB0217461		2	1 - 13 // 2 - 214	1 - 87	75	62
sigN101	DDB0185642		4	1 - 13 // 2 - 244	1 - 75	85	60

Gene Name	DDB (prediction)	DDB (curated)	Chromoso-me	Exons (exon number - bp)	Introns (intron number - bp)	Aminoacids	Identity with sigN1 protein (%)
sigN154	DDB0203952		3	1 - 13 // 2 - 136	1 - 76	49	44
sigN103	DDB0191897	DDB0230164	6	1 - 13 // 2 - 268	1 - 89	93	41
sigN109	DDB0191895	DDB0231105	6	1 - 13 // 2 - 244	1 - 120	85	40
sigN122	DDB0168559		2	1 - 13 // 2 - 199	1 - 92	70	40
sigN108	DDB0217672	DDB0219940	2	1 - 13 // 2 - 280	1 - 94	97	38
sigN137	DDB0167884		2	1 - 13 // 2 - 193	1 - 119	68	36
sigN107	DDB0191896	DDB0231563	6	1 - 13 // 2 - 268	1 - 111	93	35
sigN117	DDB0203956		3	1 - 13 // 2 - 271	1 - 85	94	34
sigN126	DDB0203951		3	1 - 13 // 2 - 280	1 - 80	97	34
sigN134	DDB0215224	DDB0216182	3	1 - 13 // 2 - 283	1 - 69	98	34
sigN144	DDB0190226		1	1 - 13 // 2 - 232	1 - 78	81	34
sigN149	DDB0215225		3	1 - 13 // 2 - 283	1 - 73	98	34
sigN153	DDB0215222	DDB0232089	3	1 - 13 // 2 - 280	1 - 76	97	34
sigN161	DDB0191898		6	1 - 13 // 2 - 187	1 - 122	66	34
sigN110	DDB0168566		2	1 - 13 // 2 - 250	1 - 110	87	33
sigN124	DDB0203948		3	1 - 13 // 2 - 280	1 - 66	97	33
sigN138	DDB0218277	DDB0232095	3	1 - 13 // 2 - 271	1 - 80	94	33
sigN151	DDB0204177		3	1 - 13 // 2 - 250	1 - 74	87	33

Gene Name	DDB (prediction)	DDB (curated)	Chromoso-me	Exons (exon number - bp)	Introns (intron number - bp)	Aminoacids	Identity with sigN1 protein (%)
sigN111	DDB0167301		2	1 - 13 // 2 - 268	1 - 116	93	32
sigN132	DDB0216937		2	1 - 10 // 2 - 220	1 -102	76	32
sigN133	DDB0215260		3	1 - 13 // 2 - 280	1 - 72	97	32
sigN150	DDB0204174		3	1 - 13 // 2 - 250	1 - 90	87	32
sigN167	DDB0205703		3	1 - 13 // 2 - 235	1 - 91	82	32
sigN112	DDB0203955		3	1 - 13 // 2 - 283	1 - 85	98	31
sigN119	DDB0218274	DDB0232102	3	1 - 13 // 2 - 286	1 - 117	99	31
sigN121	DDB0203949	DDB0232098	3	1 - 13 // 2 - 274	1 - 71	95	31
sigN146	DDB0191001		1	1 - 13 // 2 - 178	1 - 80	63	31
sigN155	DDB0204176		3	1 - 13 // 2 - 250	1 - 83	87	31
sigN113	DDB0203953		3	1 - 13 // 2 - 280	1 - 99	97	30
sigN114	DDB0215263		3	1 - 13 // 2 - 280	1 - 70	97	30
sigN120	DDB0203950		3	1 - 13 // 2 - 265	1 - 101	92	30
sigN125	DDB0218272		3	1 - 13 // 2 - 292	1 - 88	101	30
sigN148	DDB0191010		1	1 - 13 // 2 - 238	1 - 111	83	30
sigN115	DDB0215261		3	1 - 13 // 2 - 283	1 - 75	98	29
sigN116	DDB0203957	DDB0232104	3	1 - 13 // 2 - 262	1 - 74	91	29
sigN118	DDB0203954		3	1 - 13 // 2 - 271	1 - 78	94	29

Gene Name	DDB (prediction)	DDB (curated)	Chromosome	Exons (exon number - bp)	Introns (intron number - bp)	Aminoacids	Identity with sigN1 protein (%)
sigN139	DDB0187815		5	1 - 13 // 2 - 262	1 - 75	91	29
sigN141	DDB0202191		1	1 - 13 // 2 - 232	1 - 75	81	29
sigN123	DDB0218273		3	1 - 13 // 2 - 286	1 - 83	99	28
sigN128	DDB0215264		3	1 - 13 // 2 - 283	1 - 92	98	28
sigN140	DDB0189661		1	1 - 13 // 2 - 241	1 - 84	81	28
sigN142	DDB0217670		2	1 - 49 // 2 - 277	1 - 43	108	28
sigN159	DDB0204178		3	1 - 13 // 2 - 250	1 - 80	87	28
sigN160	DDB0204175	DDB0232103	3	1 - 13 // 2 - 250	1 - 96	87	28
sigN177	DDB0168238		2	1 - 13 // 2 - 262	1 - 85	91	28
sigN183	DDB0218149		3	1 - 10 // 2 - 160	1 - 85	56	28
sigN127	DDB0202241		1	1 - 103 // 2 - 253	1 - 147	118	26
sigN129	DDB0203946		3	1 - 13 // 2 - 238	1 - 84	83	26
sigN130	DDB0215262		3	1 - 303		100	26
sigN131	DDB0189832		1	1 - 13 // 2 - 250	1 - 84	87	26
sigN135	DDB0189831		1	1 - 13 // 2 - 250	1 - 97	87	26
sigN145	DDB0169368		2	1 - 13 // 2 - 223	1 - 77	78	26
sigN163	DDB0205218		3	1 - 13 // 2 - 241	1 - 79	84	26
sigN168	DDB0217669		2	1 - 13 // 2 - 262	1 - 68	91	26

Gene Name	DDB (prediction)	DDB (curated)	Chromosome	Exons (exon number - bp)	Introns (intron number - bp)	Aminoacids	Identity with sigN1 protein (%)
sigN172	DDB0218580		4	1 - 13 // 2 - 178	1 - 74	63	26
sigN166	DDB0219744		6	1 - 13 // 2 - 247	1 - 80	86	25
sigN179	DDB0189143		5	1 - 13 // 2 - 214	1 - 79	75	25
sigN182	DDB0168809		2	1 - 13 // 2 - 250	1 - 109	87	25
sigN156	DDB0191875		6	1 - 13 // 2 - 259	1 - 80	90	24
sigN143	DDB0169369		2	1 - 13 // 2 - 223	1 - 114	78	23
sigN152	DDB0192087		6	1 - 13 // 2 - 253	1 - 73	88	22
sigN157	DDB0189829		1	1 - 13 // 2 - 259	1 - 86	90	22
sigN158	DDB0202029		1	1 - 49 // 2 - 229	1 - 38	92	22
sigN162	DDB0190588		1	1 - 13 // 2 - 262	1 - 85	91	22
sigN169	DDB0190590		1	1 - 13 // 2 - 262	1 - 85	91	22
sigN171	DDB0191873		6	1 - 13 // 2 - 229	1 - 98	80	22
sigN174	DDB0204123		3	1 - 13 // 2 - 220	1 - 84	77	22
sigN175	DDB0187094		4	1 - 13 // 2 - 190	1 - 89	67	22
sigN164	DDB0187084		4	1 - 13 // 2 - 253	1 - 107	88	21
sigN165	DDB0169119	DDB0229924	2	1 - 13 // 2 - 250	1 - 91	87	21
sigN136	DDB0202240		1	1 - 13 // 2 - 253	1 - 128	88	20
sigN170	DDB0205706		3	1 - 13 // 2 - 244	1 - 91	85	20

Gene Name	DDB (prediction)	DDB (curated)	Chromoso-me	Exons (exon number - bp)	Introns (intron number - bp)	Aminoacids	Identity with sigN1 protein (%)
sigN147	DDB0191871		6	1 - 13 // 2 - 274	1 - 89	95	19
sigN173	DDB0215631		4	1 - 13 // 2 - 181	1 - 73	64	17
sigN176	DDB0185480		4	1 - 10 // 2 - 178	1 - 93	62	17
sigN178	DDB0187095		4	1 - 13 // 2 - 118	1 - 88	43	16
sigN181	DDB0215630		4	1 - 13 // 2 - 181	1 - 71	64	14
sigN180	DDB0189205		5	1 - 13 // 2 - 205	1 - 110	72	12
